# Gaze Behavior During Navigation and Visual Search of an Open-World Virtual Environment

**DOI:** 10.3389/fpsyg.2021.681042

**Published:** 2021-08-09

**Authors:** Leah R. Enders, Robert J. Smith, Stephen M. Gordon, Anthony J. Ries, Jonathan Touryan

**Affiliations:** ^1^DCS Corp., Alexandria, VA, United States; ^2^DEVCOM Army Research Laboratory, Aberdeen Proving Ground, MD, United States; ^3^Warfighter Effectiveness Research Center, U.S. Air Force Academy, Colorado Springs, CO, United States

**Keywords:** visual search, virtual environment, eye tracking, distractors, dwell time, divided attention

## Abstract

Eye tracking has been an essential tool within the vision science community for many years. However, the majority of studies involving eye-tracking technology employ a relatively passive approach through the use of static imagery, prescribed motion, or video stimuli. This is in contrast to our everyday interaction with the natural world where we navigate our environment while actively seeking and using task-relevant visual information. For this reason, an increasing number of vision researchers are employing virtual environment platforms, which offer interactive, realistic visual environments while maintaining a substantial level of experimental control. Here, we recorded eye movement behavior while subjects freely navigated through a rich, open-world virtual environment. Within this environment, subjects completed a visual search task where they were asked to find and count occurrence of specific targets among numerous distractor items. We assigned each participant into one of four target conditions: Humvees, motorcycles, aircraft, or furniture. Our results show a statistically significant relationship between gaze behavior and target objects across Target Conditions with increased visual attention toward assigned targets. Specifically, we see an increase in the number of fixations and an increase in dwell time on target relative to distractor objects. In addition, we included a divided attention task to investigate how search changed with the addition of a secondary task. With increased cognitive load, subjects slowed their speed, decreased gaze on objects, and increased the number of objects scanned in the environment. Overall, our results confirm previous findings and support that complex virtual environments can be used for active visual search experimentation, maintaining a high level of precision in the quantification of gaze information and visual attention. This study contributes to our understanding of how individuals search for information in a naturalistic (open-world) virtual environment. Likewise, our paradigm provides an intriguing look into the heterogeneity of individual behaviors when completing an un-timed visual search task while actively navigating.

## Introduction

Active, unconstrained visual exploration is the sensory foundation of how the majority of individuals interact with the natural world, continually seeking information from their environment. This often includes coordinated body, head, and eye movement activity. In contrast, the majority of studies that seek to understand human visual perception employ a relatively passive approach through the presentation of stimuli, whether synthetic or natural. Likewise, body, head, and even eye movements are often constrained, either explicitly or by the nature of the experimental paradigm. These factors help control the manifold sources of variability, enabling the meaningful interpretation of finite empirical data. However, as both our understanding of perception and experimental capabilities expand, an increasing number of studies have sought to explore visual processes under more natural conditions; enhancing ecological validity while maintaining construct validity (Diaz et al., 2013; Foulsham and Kingstone, 2017).

Here, we build upon a body of work that has used virtual environments to understand perceptual and cognitive processes related to visual search and navigation. Previous work investigating visual search in virtual environments have examined eye movements during object search and memory tasks (Draschkow et al., 2014; Kit et al., 2014; Li et al., 2016; Helbing et al., 2020) and have examined visual attention toward distractors (Olk et al., 2018) using timed task paradigms in traditional indoor virtual environments where items are placed in-context with surrounding environments. Previous work in the areas of spatial cognition and navigation, have created virtual maze environments to investigate visual attention during employment of allocentric and egocentric navigation strategies (Livingstone-Lee et al., 2011) and to understand the role of gender in landmark utilization (Andersen et al., 2012). In addition, virtual environments have been used to test the effectiveness of a guidance system during navigation of a train station (Schrom-Feiertag et al., 2017), and to examine spatial knowledge (Clay et al., 2019) and change detection (Karacan et al., 2010) during navigation of outdoor virtual environments. Other predecessors in this area of work have laid the ground work to integrate open sourced game engines with eye tracking to create naturalistic environments for multimodal neurophysiological research (Jangraw et al., 2014). Despite these efforts, additional work is needed to understand how visual search generalizes in a variety of real-world contexts, such as navigation and how targets are found during navigation with limited spatial (contextual) dependencies. Likewise, the capability to link gaze behavior in these complex environments, to neurophysiological processes, remains an open challenge for the field.

Measuring eye movement activity, including saccades, fixations, and blinks, has provided researchers a non-invasive way to gain valuable insight into perceptual, attentional, and cognitive processes during visual search tasks (Hoffman and Subramaniam, 1995; Kowler et al., 1995; Deubel and Schneider, 1996; Williams and Castelhano, 2019). Examining fixation metrics (e.g., number of fixations or dwell time) can indicate how individuals process visual information. For example, previous work has shown that individuals increase the number of fixations and dwell time (summation of all individual fixation durations) on informative visual objects within a scene (Loftus and Mackworth, 1978) and in the detection of changes of an object's location within a scene (Võ et al., 2010). Improved memory recall and recognition on tasks is associated with increased number of fixations (Kafkas and Montaldi, 2011; Tatler and Tatler, 2013) and increased dwell time (Hollingworth and Henderson, 2002; Draschkow et al., 2014; Helbing et al., 2020). Specifically, increased number of fixations and increased dwell time on objects during visual search tasks are linked to improved memory for those objects (Hollingworth and Henderson, 2002; Tatler and Tatler, 2013; Draschkow et al., 2014; Helbing et al., 2020). This also appears to be the case when comparing how individuals visually attend to target objects compared to distractors in the environment. Horstmann et al. (2019) found that the average number of fixations on visual targets (about 1.55) was higher compared to the average number of fixations on similar looking distractors (about 1.20) during a search task with static images. Watson et al. (2019) reported that the number of fixations on targets ranged from about 3.3–4 compared to around 2.8–3.8 fixations on distractors, during a free visual search, and reward learning task in a virtual environment. In terms of dwell time, Draschkow et al. (2014) found subjects looked about 0.6 s longer at targets as compared to distractors during visual search of static natural scenes.

Previous work suggests that visual search tasks using traditional stimuli such as static pictures may yield different findings than those incorporating real world scenarios (Kingstone et al., 2003). Research has shown notable differences in gaze metrics between simple static vs. complex dynamic visual search tasks, arguing for the increasing utilization of dynamic scenes. For instance, Smith and Mital (2013) found increased dwell time on visual objects and increased saccade amplitude during a viewing and identification task in a dynamic scene compared to a static scene. We live in a visually complex world that includes many visual points of interest, depth, motion, and contextual scene information. Therefore, real-life environments are seemingly the optimal stimuli to study naturalistic eye movement during visual search.

To this end, researchers have employed free navigation visual tasks in real-life scenarios such as walking outdoors (Foulsham et al., 2011; Davoudian and Raynham, 2012; Matthis et al., 2018; Liao et al., 2019), walking indoors (Kothari et al., 2020), driving (Land and Lee, 1994; Dukic et al., 2013; Grüner and Ansorge, 2017; Lappi et al., 2017), and shopping in a grocery store (Gidlöf et al., 2013), to name a few. Although eye tracking in a real-life scenario allows free body movement, conducting studies in real environments can be difficult if not impossible to control; every subject's unique actions makes a comparative analysis difficult. Fotios et al. (2015) noted this challenge in a study that examined eye movement for pedestrians walking down the street. Examining eye movement metrics in real life environments also limits the design of the study in terms of the availability of targets and distractors (i.e., extant objects or limited by budget) and may be limited on the ability to gather neurophysiological measures such as electroencephalogram (EEG) recordings. Furthermore, real-world paradigms are often limited to only locally accessible environments and restrict researchers from studying more consequential scenarios where there are high demands for visual attention during a search task (e.g., looking for threat targets in a combat zone).

The use of virtual environments in perception research is an ecologically valid approach that provides the ability to conduct studies in an interactive but controlled dynamic environment (Parsons, 2015). Since eye-tracking systems can now be readily integrated with 3D rendering software (i.e., game engines), researchers can conduct eye movement studies in more realistic and immersive environments (Watson et al., 2019). Virtual environments also allow for research designs that may otherwise not be practical for a real-world implementation. For example, Karacan et al. (2010) utilized a 3D rendered virtual environment to examine shifts in gaze patterns as subjects repeatedly walked a loop path looking for isolated changes in the environment during each lap (e.g., a new object appearing, changing, and/or disappearing). The use of the virtual environment allowed for uninterrupted “physical” and visual inspection of an environment with tightly controlled visual changes. Virtual environments can accommodate research in attentional control and even allow for quantifiable interactions with objects in the scene. Helbing et al. (2020) utilized a virtual reality environment to examine memory encoding during target search of 10 different complex and naturalistic indoor rooms. Furthermore, utilizing game engines as Unity3D (Unity Technologies), can allow for the subjects to remain stationary during visual exploration of an environment and for researchers to perform synchronous acquisition of multiple physiological modalities, including respiration, electrocardiography (EKG), and EEG (Jangraw et al., 2014) that would otherwise be difficult in an ambulatory condition.

Similar to previous work in our field, the current study seeks to isolate distinct gaze behaviors associated with target objects during an active visual search of a complex environment. Here, subjects freely navigate through a virtual world while completing a self-paced visual search task identifying assigned targets placed amongst many distractors (all other objects in the virtual environment other than targets). These distractors include a wide variety of objects that are, in some cases, similar in shape, or color to the assigned target object. Additionally, objects in the world appear to be inconsistently placed along the path (e.g., laying on their side or standing upright) and randomly placed among one another with little to no scene context in regards to surrounding items or, in some cases, to the environment itself (e.g., a tuba next to a washing machine next to an airplane). This is particularly interesting since more traditional work with static scenes, has shown the importance of scene context on eye movement, memory, and search time for visual targets (Loftus and Mackworth, 1978; Henderson et al., 1999; Castelhano and Heaven, 2010; Draschkow et al., 2014). Lastly, some of the subjects are assigned a Target Condition that includes more than one particular target object in the environment. This study also includes an auditory divided attention task to increase subjects' cognitive load during a portion of the visual search task, which enables us to further investigate how subjects compensate visual attention during a self-paced task in a complex environment.

The primary aim of this study is to quantify visual search behavior during navigation of an open virtual environment and identify similarities and differences between related work that used more traditional fixed, static scenes. To this end, we quantify the difference in gaze metrics between task-relevant targets and task-irrelevant distractors (that do not provide context for locating a target) and during high and low cognitive load conditions, comparing the results to previous studies which utilized more traditional visual search and encoding paradigms. Specifically, we expect there to be an increased number of fixations and dwell time on targets, as compared to distractors (Draschkow et al., 2014; Horstmann et al., 2019; Watson et al., 2019). We likewise expect subjects will visually explore targets at a closer distance as compared to other objects in the environment. Finally, we anticipate that auditory math task will elicit changes in saccade or fixation activity, such as increased visual attention on scanned objects in the environment (Pomplun et al., 2001; King, 2009; Buettner, 2013; Zagermann et al., 2018) or a change in exploratory behavior (i.e., reduction in speed and number of objects viewed) of the environment, due to increased cognitive load.

## Materials and Methods

### Subjects

Forty-Five subjects, recruited from the Los Angeles area, participated in this study [17 females with mean age ± standard deviation (*SD*) = 36.8 ± 12.3 years, 28 males with mean age ± *SD* = 41.6 ± 14.4 years]. All subjects were at least 18 years of age or older and able to speak, read, and write English. All subjects signed an Institutional Review Board approved informed consent form prior to participation (ARL 19–122) and were compensated for their time. All subjects had normal hearing and normal or corrected-to-normal vision and had normal color vision. All subjects completed a web-based pre-screen questionnaire containing eligibility, demographic, and game-use questions. Additional color vision and visual acuity screening was conducted in-lab to ensure a minimum of 20/40 vision, using a standard Snellen Chart, and normal color vision, assessed with a 14-plate Ishihara color test. Any subject who did not pass the screening process was not included in the study. Subjects completed a simulator sickness screening questionnaire, the Simulator Sickness Questionnaire (SSQ) (Kennedy et al., 1993), before and after Pre-Test training (see below) and then again after the main study task. The mean and SD for the Total Weighted Score from the SSQ was 12.6 ± 16.4 before the system training task, 32.1 ± 30.7 after the training task, and 38.1 ± 37.5 after the main study task. As part of the questionnaire, subjects answered questions relating to video game experiences and weekly usage of video games. The average number of years playing video games was 28.0 ± 11.4 years. The mean age when subjects began playing video games was 12.8 ± 7.1 years. Over half of subjects (51%) reported playing video games <2 h a week. Almost a third (29%) of the subjects reported playing video games 2–7 h a week. The remaining 20% of subjects reported playing video games for >8 h a week. Six subjects were later removed from the main study analysis (*N* = 39) and an additional one subject was not included in the Math Task analysis (*N* = 38) due to reasons detailed below.

### Procedure

During the experimental session, subjects participated in four separate tasks: a go/no-go serial visual presentation, an old/new recognition task, and two virtual environment tasks. However, only results from the virtual environment training and navigation tasks are described here. The stimuli in the other tasks were unrelated to the virtual environment.

#### Overview

**Figure 1:**
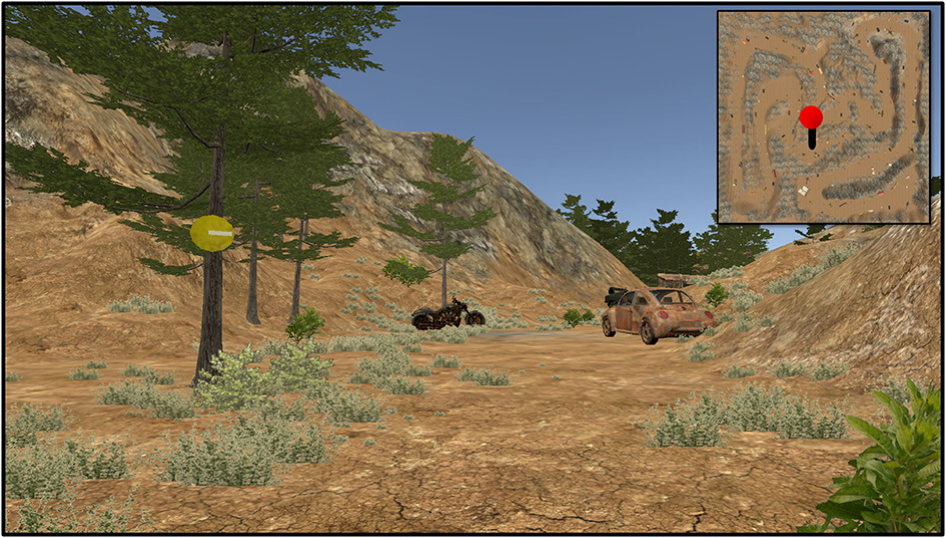
First person point of view near the beginning of the task. Trail makers (yellow circles with direction indicator) were placed on trees throughout the environment. Targets were assigned to each Target Condition to count: Humvee, motorcycle (shown), aircraft, and furniture. Distractors were any object in the environment not assigned to the subject (e.g., tires, dumpster, Humvees for anyone not in the Humvee Condition). Inset (not visible during experiment) shows the current position on the complete map.

Subjects were asked to freely navigate the virtual environment with the goal of searching for and counting their assigned target objects. All subjects were randomly assigned to one of four Target Conditions: Humvee Condition (*N* = 15 subjects), Motorcycle Condition (*N* = 14 subjects), Aircraft Condition (*N* = 9 subjects), or Furniture Condition (*N* = 7 subjects). The Aircraft and Furniture Conditions were introduced later in the data collection, which was eventually halted due to restrictions on in-person studies, hence the lower subject numbers. The aircraft and furniture targets were already present in the environment prior to introduction of the two new Conditions, thus, all subjects in every Target Condition navigated the same environment with the same objects in the same order (Figure 1). Natural landscape features and trail markers provided a suggested path through the virtual environment (although subjects could freely explore in any chosen direction).

#### System Training Task

A training task was used to acclimate subjects to navigation in the virtual environment via the keyboard and mouse. Movement was controlled with the W/A/S/D keys: “W” moved the subject in the forward direction, “A” allowed the subject to move left, “S” moved the subject backwards, and “D” allowed the subject to move right. A computer mouse was used to control the camera orientation or viewport (i.e., first person perspective) while in the virtual environment. This training environment was similar to the virtual environment used during the main task but contained different objects. This training task also ensured subjects were not acutely susceptible to simulator sickness.

#### Testing Setup

The experimental setup for this study combined multiple physiological modalities: eye-tracking, EEG, electrocardiography (EKG). Here, we described the relationship between task features, performance, and eye movement behavior. Other modalities, such as EEG and EKG, will be discussed in future reports and are not included in the current study.

All tasks were run using custom software built in the Unity 3D environment (Unity Technologies) run on the standard Tobii Pro Spectrum monitor (EIZO FlexScan EV2451) with a resolution of 1,920 x 1,080 pixels. Subjects were seated at a distance of ~70 cm from the monitor. Eye tracking data were collected with a Tobii Pro Spectrum (300 Hz). In addition to obtaining gaze position and pupil size, the Tobii Pro SDK was used to calculate the 3D gaze vector (invisible ray representing the instantaneous gaze direction) and identify the gaze vector collision object (first object in the Unity environment that collides with the 3D gaze vector) for each valid sample. The eye tracking data were synchronized with the game state, keyboard, mouse, and EEG data using the Lab Streaming Layer protocol (Kothe, 2014). A standard 5-point calibration protocol was used to calibrate the eye tracker. The Tobii Pro Spectrum has an average binocular accuracy of 0.3°, binocular precision (root mean square) of 0.07°, and detects 98.8% of gazes (Tobii Pro, 2018). However, no verification of these error metrics was performed for this study. Head movement was not restricted in terms of head support or a chin rest. However, subjects were asked to maintain an upright, yet comfortable posture to minimize large upper body movements and maintain proper alignment with the eye tracker.

#### Virtual Environment Description

**Figure 2:**
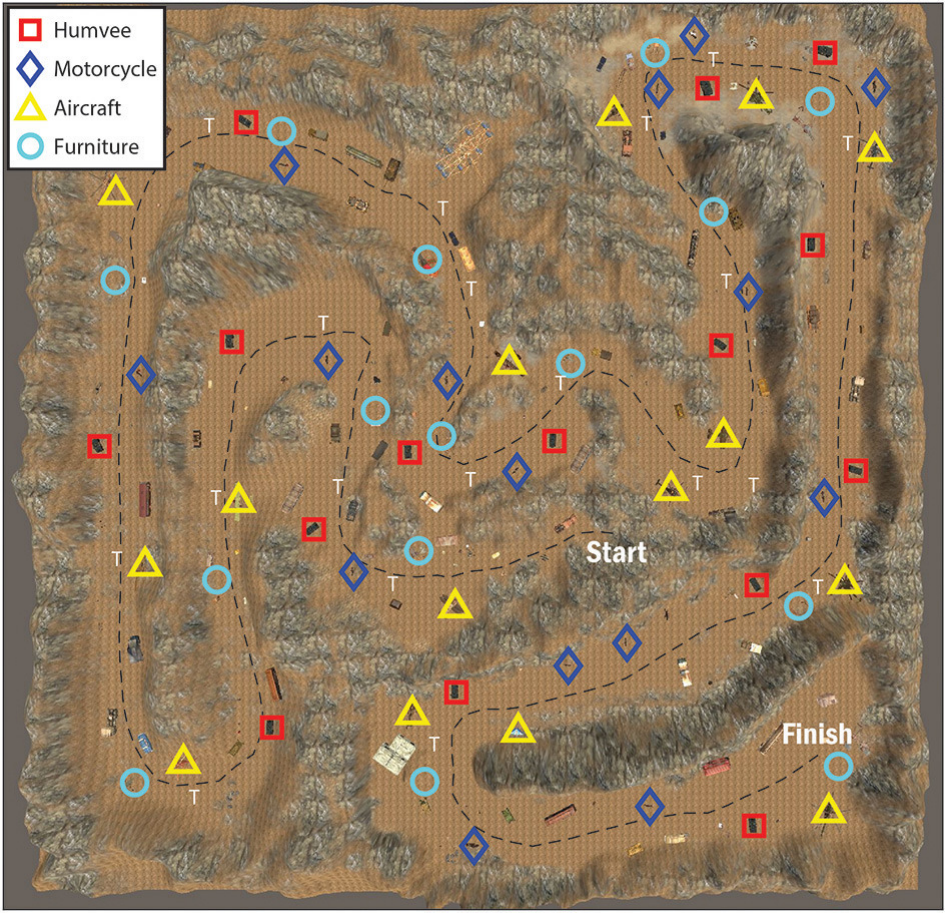
General layout of the virtual environment map. The black checkered line represents an example subject's path from the starting area to the finish. Target icons are as follows: furniture (light blue circle), motorcycle (dark blue diamond), aircraft (yellow triangle), and Humvee (red square). Trail markers are present throughout the path and are indicated by a white “T.”

**Figure 3:**
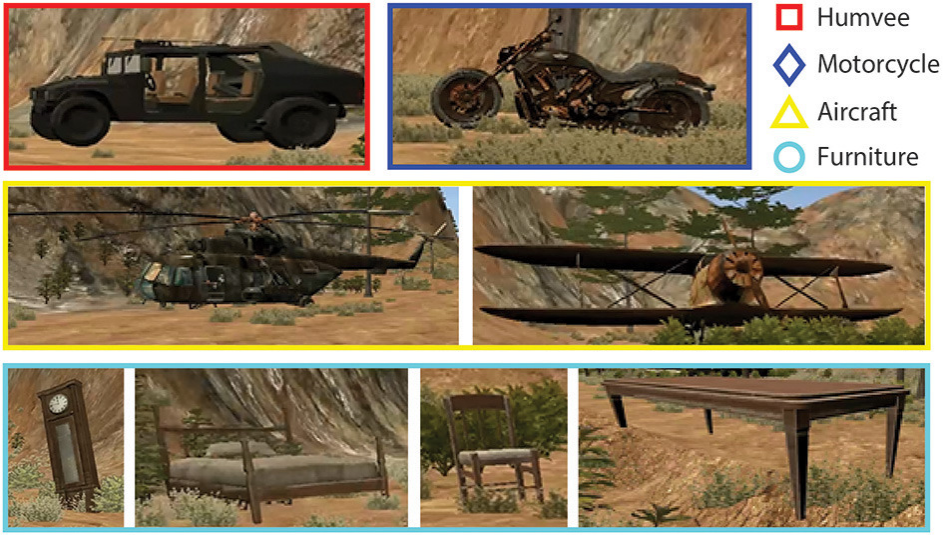
Example pictures of the targets for each Target Condition as they appear in the virtual environment. Motorcycles and Humvees (top row) did not vary in model but did vary in how they were positioned on the trail (i.e., motorcycle against a tree or at an angle by a rock). Both aircrafts (middle row) and furniture (third row) targets varied in shape, size, and positioning on the trail.

Targets were placed in a random sequence at semi-regular intervals along the path in the virtual environment. The location of all targets and objects in the environment were the same for all subjects. A general layout of the environment, indicating all target locations, is shown in Figure 2 and target examples are shown in Figure 3. As stated previously, all objects were embedded in the environment in such a way that each appeared randomly placed with no context gained from neighboring objects. Thus, targets (and distractors) appeared along the path and could not be anticipated by surrounding objects that may give the subjects an indication that a target was visually missed, present, or forthcoming. Subjects all started at the same point on the virtual environment. Trail markers (*N* = 19) were placed along the trail for general navigational guidance. There were 15 targets total for each Target Condition. The same model of Humvee was used for all the *Humvee targets* and the same model of motorcycle was used for the *motorcycle targets*. For the *aircraft targets*, models varied and included helicopters, bi-planes, and one glider. For the *furniture targets*, objects included variations such as beds, grandfather clocks, tables, and a variety of seating furniture (e.g., sofa, dining chair). Sizes varied for the furniture with the chair being the smallest and the bed being the largest furniture target. Around 166 additional objects were included in the virtual environment that were not an assigned target to any Target Condition. These additional objects included, but were not limited to, cars, trucks, tanks, an oven, a drum set, a Ferris wheel, a pile of tires, dumpsters, and shipping containers. For analysis, a distractor was defined as any visual object in the environment not belonging to the specified Target Condition and included objects assigned as targets to other Target Conditions (e.g., Humvees were considered distractors for the Motorcycle Condition). Terrain (e.g., trees, hillside, grass, path) and the sky were not included in the analysis unless explicitly mentioned.

#### Subject Instruction and Navigation

Subjects were instructed to search and count (mentally) when they saw a target assigned to their Target Condition. Subjects were encouraged to stay on or near the trail (and at times were verbally reminded by research staff) to make sure they encountered all objects, but were free to navigate as desired. Midway (8 min) into the session an auditory Math Task (divided attention task) was administered (see below for details). Subjects had up to 20 min to progress through the virtual environment and reach the finish. If subjects did not complete the task in 20 min, and if they did not encounter (as determined by their gaze vectors) at least 10 targets in the virtual environment, then their data was removed from statistical analysis. For this reason, data from two people in the Furniture Condition were removed from all analysis. In addition, one subject in the Humvee Condition reported feeling unwell during testing and experienced difficulty in navigating the environment (i.e., did not follow the path) and thus, their data was also removed from the analysis. The average time to complete navigation of the virtual environment was about 12 (± 2) min. After completion, subjects were asked to recall how many targets they saw during the navigation task.

#### Additional Math Task

Starting at the 8 min mark, an auditory math problem was presented to the subjects. An auditory recording of a set of 3 to 4 numbers, with values between 0 and 9, was played for the subject through headphones (e.g., “4,” pause, “2,” pause, “8,” tone, subject reports “14”). A pause of 3–4 s separated each number in a set, and each set was followed by a tone. After the tone, subjects verbally reported the sum of numbers aloud to the experimenter. During the Math Task, subjects were instructed to continue navigating through the virtual environment and continue searching and mentally counting their targets. This Math Task was repeated two more times (with different sets of numbers), for a total of three summation responses. There was an 8–30 s break between each set of numbers. Because the primary search task was self-paced, it is possible that a subject would finish exploring the virtual environment (reach the end of the path) without completing the Math Task. Only one subject (in the Humvee Condition) did not complete the Math Task prior to finishing the navigation task and for this reason, their data was removed from the Math Task analysis.

### Data Extraction and Analysis

#### Fixation Detection and Object Labeling

**Figure 4:**
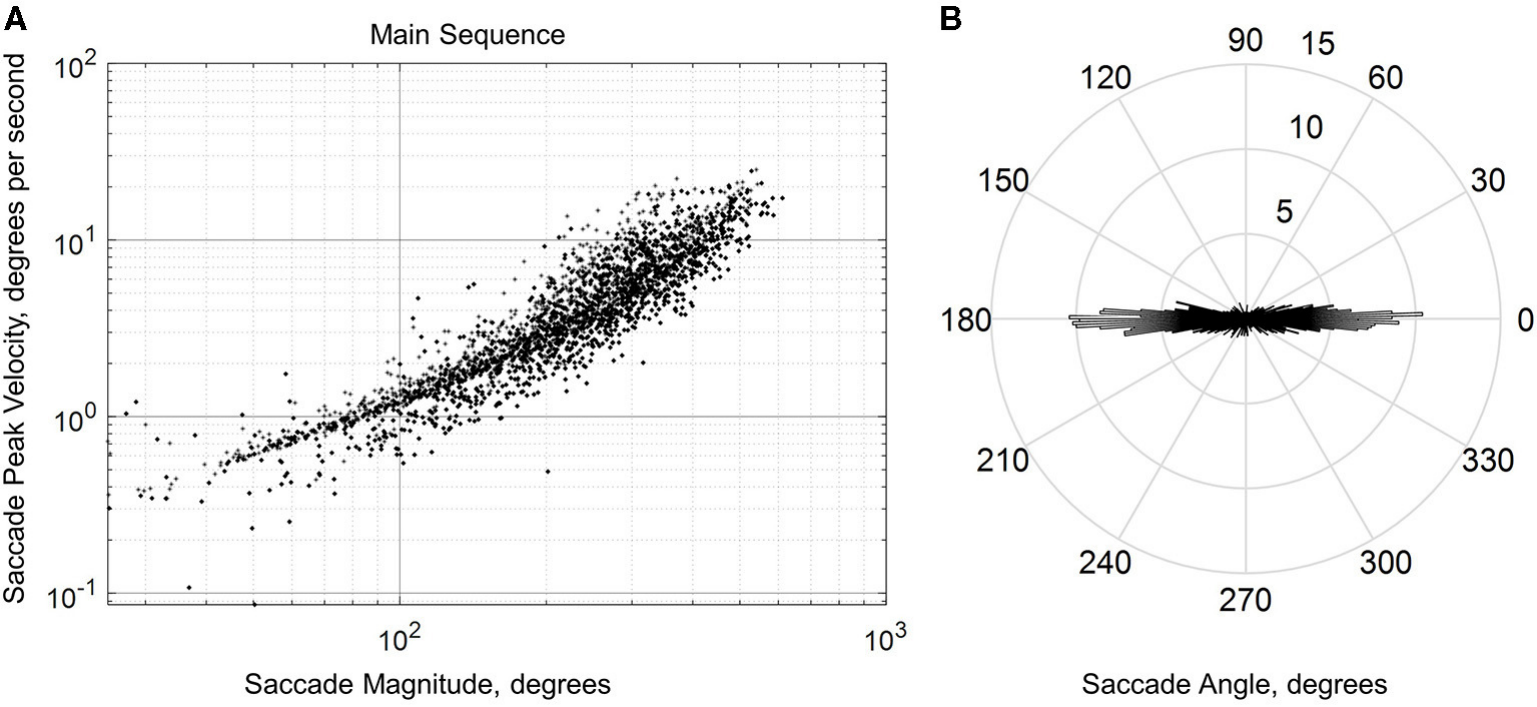
Saccade main sequence (in log-log coordinates) **(A)** and angle distribution **(B)** from a sample of the first 500 saccades from five representative subjects. The saccade angle distribution histogram has a one-degree resolution, with the radial axis showing average number and the angular axis showing average angle across all five subjects.

**Figure 5:**
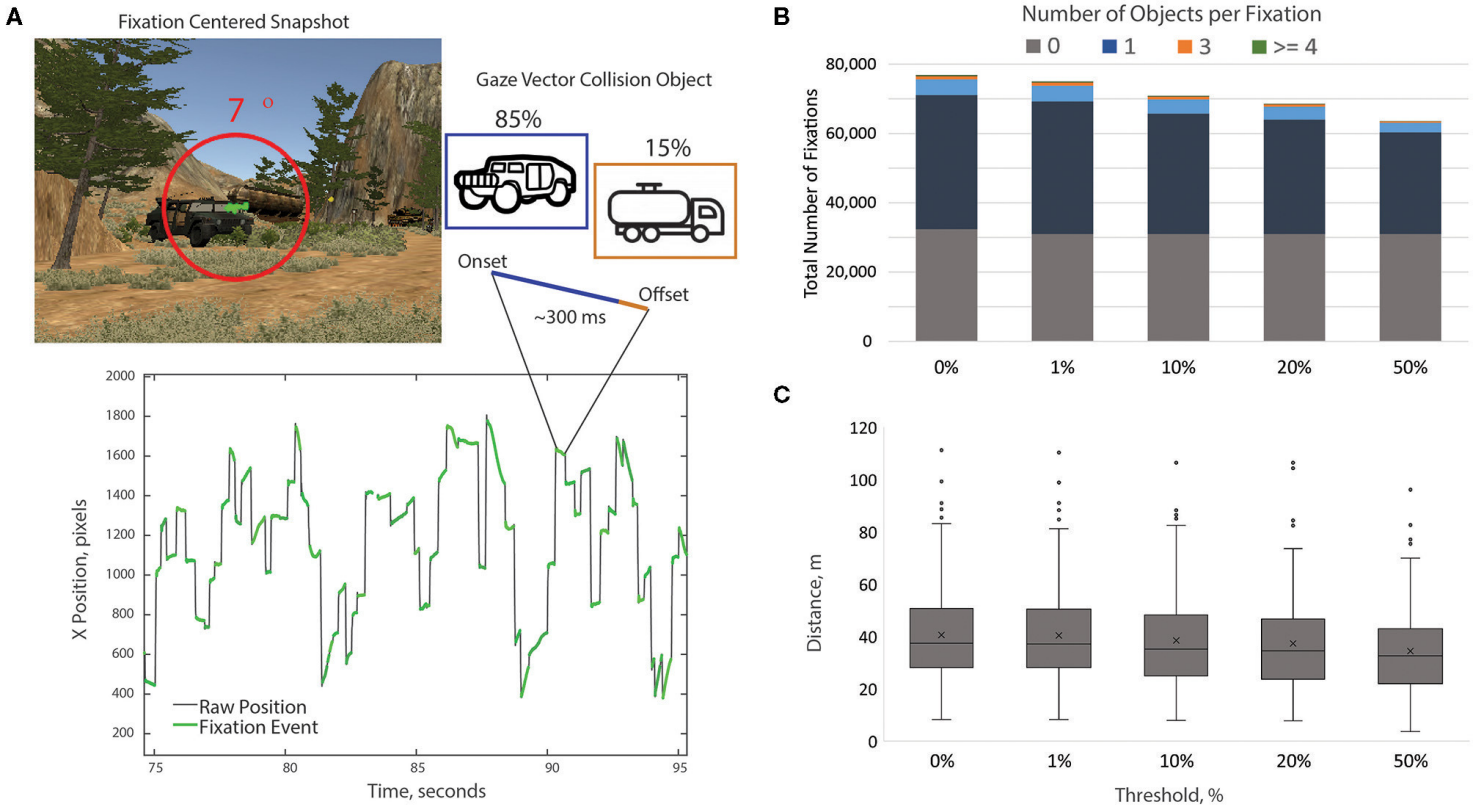
Fixation labeling approach. Saccades and fixations are detected from the raw X/Y gaze position time series. Note, that the inter-saccadic intervals (fixations) exhibit a range of velocities and include stable fixations, smooth pursuit movements, and optokinetic responses. However, these velocities were significantly lower than the ballistic threshold of the saccade detection algorithm. Each valid gaze sample is associated with an object via gaze vector collision. Fixations are then associated with objects if the dominant object (excluding terrain and sky) is contained in at least 10% of the samples of the epoch. **(A)** Alternative thresholds were examined and the 10% threshold appeared to filter out erroneous labels without excluding large amounts of meaningful data. The impact of the threshold value on the number of **(B)** and distance to **(C)** objects with all fixation epochs.

Blinks were identified from stereotyped gaps in the gaze position data (Holmqvist et al., 2011) while saccades (and corresponding fixations) were detected using a standard velocity-based algorithm (Engbert and Kliegl, 2003; Engbert and Mergenthaler, 2006; Dimigen et al., 2011) adapted from the EYE-EEG plugin (http://www2.hu-berlin.de/eyetracking-eeg). Specifically, velocity thresholds for saccade detection were based on the median of the velocity time series, smoothed over a 5-sample window, for each subject. Thresholds were computed independently for horizontal and vertical components. In this study, we used a velocity factor of six (six times the median velocity), a minimum saccade duration of 12 ms, and a minimum fixation duration (i.e., inter-saccadic interval) of 50 ms. We kept only the largest saccade and subsequent fixation if two or more saccades were detected within the minimum fixation duration window. Visual inspection of the saccades shows the expected relationship between saccade peak velocity and saccade magnitude (i.e., main sequence; Figure 4A). Only the first 500 saccades of 5 subjects are shown in this figure due to the large amount of saccades generated by each subject (~8–20 min of eye tracking). These 5 subjects were chosen randomly and are representative of the entire subject data set. These saccade distributions excluded blinks, dropouts, saccades with a duration shorter than 12 and >100 ms, and peak velocities outside of a range of 25 and 1,200 degrees per second. The distribution of the saccade angle showed a strong tendency for subjects to scan the horizon (Figure 4B). Finally, blinks were defined as gaps with a duration ranging from 50 to 500 ms and dropouts were defined as any gap with a duration <50 or >500 ms (any gap not considered a blink). While the detection algorithm used in this study was developed for ballistic saccades, visual inspection revealed that it was reasonably successful at separating saccades from other eye movement features such as smooth pursuit and optokinetic responses (Figure 5A).

After initial saccade detection, fixations of <100 ms were discarded and not used in any subsequent analysis (Ouerhani et al., 2004; Mueller et al., 2008; Andersen et al., 2012). In addition to standard metrics associated with fixations (e.g., duration), each fixation was assigned a virtual environment object label using the following approach. Every valid gaze sample returned a corresponding object that was the result of the gaze vector collision. The object with the highest percentage of collisions over the fixation epoch was assigned as the “fixation object” (Figure 5A). Target fixations were labeled as such if the highest percentage of collisions were on a target (e.g., motorcycles for the Motorcycle Condition) and this number amounted to at least 10% of all gaze samples for that fixation. Distractor fixations were labeled using the same metric; receiving a distractor designation label if at least 10% of the gaze samples included the same distractor object. This 10% threshold was utilized to identify the primary fixation object and reduce the chance of including adjacent or background objects. Objects could be erroneously included in a fixation epoch from either gaze vector estimation error or having a relative position directly behind the primary fixation object. We selected the 10% value by assessing fixation labels at a range of thresholds: 0, 1, 10, 20, and 50% (Figures 5B,C). The 10% threshold appeared to be a middle ground between reducing the chance of erroneous fixations without removing a large number of meaningful fixations. Fixation data from three subjects (two from the Aircraft Condition and one from the Humvee Condition) had a high dropout rate (high number of invalid samples). Thus, these three subjects were removed from the analysis.

#### Calculation of Fixation Variables for the Main Study Analysis

From the fixation data for the targets, the following variables in Table 1, were calculated. The Self-Reported Target Count and the Gaze-Validated Target Count (the instances where the ray cast at the fixation intersected with the object using the above fixation labeling approach) were calculated to compare subjective inventory with detected target fixations. To identify if our approach was sensitive enough to detect increased visual attention on targets, the Mean Number of Fixations, Mean Dwell Time, and Mean Distance were compared. Distance is included to provide a relative measure of how “close” subjects approached objects in the environment. Importantly, although the units here are given in meters, we acknowledge that this metric is not an equivalent analog to the real world (i.e., meters in the virtual environment may not reflect an actual meter in real life). Variation in object size and structural diversity impacted these particular fixation metrics. For instance, object surface area in the virtual environment was shown to be a large covariate with Mean Number of Fixations (Spearman's rho = 0.719, *p* = 0.000), Mean Dwell Time (Spearman's rho = 0.630, *p* = 0.000), and Mean Distance (Spearman's rho = 0.558, *p* = 0.000). The larger the object, the increased chance a subject has to see it at any given viewing point, regardless of attentional focus. To help account for this bias, three additional variables, Normalized Number of Fixations, Normalized Dwell Time, and Normalized Distance were calculated utilizing the Global Number of Fixations, Global Dwell Time, and Global Distance variables. The global values were then used to normalize the means associated with the diversity of Target for each Target Condition. Because of the large size disparity between objects in the virtual environment, normalization by dividing gaze data by object size (utilizing either the 3D volume or 2D profile) resulted in a large bias toward the smaller targets.

**Table 1:** Dependent and independent variable list and definitions.

**Variable**	**Definition**
Target condition	Each subject was randomly assigned to one of four groups, named for the target they were assigned (i.e., Humvee Group assigned to look for Humvee targets)
Self-reported target count	Total count of targets that the subject reported seeing during exploration of the virtual environment
Gaze-validated target count	Total number of targets having at least one qualifying fixation associated with that target
Mean number of fixations	Average number of qualifying fixations per each object
Mean dwell time	Average total duration of fixations per each object
Mean distance	Average distance from the object when each associated fixation occurred
Global number of fixations	Average number of fixations for that particular object across all subjects
Global dwell time	Average dwell time for that particular object across all subjects
Global distance	Average distance from where that object was fixated across all subjects
Normalized number of fixations	Mean Number of Fixations subtracted from the Global Number of Fixations
Normalized dwell time	Mean Dwell Time subtracted from the Global Dwell Time
Normalized distance	Mean Distance subtracted from the Global Distance
Mean duration of individual fixations	Average of all individual fixations across all Target Conditions and objects (targets and distractors) in the environment
Fixation rate	Summation of all fixations during the Math Task (or outside of the Math Task) divided by the total time spent in that time period
Object rate	Total number of distinct objects that were fixated per unit time
Blink rate	Summation of all blinks during the Math Task (or outside of the Math Task) divided by the total time spent in that time period
Proportion of fixations on objects	Summation of fixations on objects (as opposed to terrain or sky) divided by sum of all fixations overall
Position velocity	Average change in position over time

As an additional analysis, we included a comparison of gaze data between just the Humvee and Motorcycle Conditions to identify differences in gaze behavior between the Humvee and motorcycle objects. This analysis provided evidence of how subjects in two different Target Conditions examined these two particular objects differently and how target assignment impacted gaze metrics. The Humvee and the Motorcycle Conditions were utilized in this way because these two conditions were comparable in subject numbers (*N* = 13 and 14, respectively) and target attributes (i.e., same object model throughout the environment). In addition, these two conditions had targets that differed greatly in size and, as previously stated, we expected there to be differences in non-normalized gaze metrics, simply due to size of the object alone.

For the Math Task, fixation data from the following two time periods was compared: outside (before and after) and during the Math Task. To see if subjects changed the rate at which they fixated objects due to the Math Task, the Mean Duration of Individual Fixations *on objects* and Fixation Rate were compared between these time periods. To determine if subjects compensated for divided attention during the Math Task by reducing the overall amount of visual attention devoted to each object, the Mean Number of Fixations *per each object* and Mean Dwell Time *per each object* was compared between the two time periods. Object Rate, the number of distinct objects that were fixated per unit time, was compared across the two time periods to capture differences in visual scanning behavior. To understand if subjects compensated for divided attention during the Math Task by reducing visual attention on particular objects and instead focused on background scenery, the Proportion of Fixations on Objects was compared between the two time periods. To examine if subjects speed up or slowed down their navigating through the environment, the Position Velocity was compared between the two time periods. Lastly, Blink Rate examined if subjects changed the number of blinks per unit of time with increased cognitive load.

### Statistical Analysis

To summarize, data from three subjects were removed due a high dropout rate, data from two subjects were removed due to not encountering the minimum threshold of targets, and data from one subject (who reported feeling ill during the testing) had navigational issues was removed, bringing the final inclusion of *N* = 39 subjects for analysis. In addition, one subject finished navigating the environment prior to the completion of the Math Task, for a total of *N* = 38 for that analysis. For the remaining subjects', a normal distribution was assessed for all fixation variables using Kolmogorov-Smirnov and Shapiro-Wilk tests for normality. Parametric tests (i.e., Paired Samples *t*-test, MANOVA) were used for variables with normal distributions and non-parametric tests (i.e., Related-Samples Wilcoxon Signed Rank Test, Friedman Test) for non-normal distributions. For this reason, non-parametric statistical methods were utilized for the measures of Self-Reported Target Count, the Gaze-Validated Target Count, Blink Rate, and Position Velocity. All other variables had a normal distribution and parametric tests were used for comparative analysis. Outliers in the data were designated as samples/observations that were greater or less than three standard deviations from the mean. Outliers were removed from the data prior to analysis and includes one person's data for Mean Distance and Normalized Dwell Time (*N* = 38 for analysis with these measures) and one person's data for Mean Duration of Individual Fixations *on objects*, Mean Dwell Time *per object*, and Blink Rate during the Math Task analysis (*N* = 37 for analysis with these measures). In addition, Self-Reported Target Count was missing for six additional individuals (who did not report an answer when prompted) and one outlier was removed from the Self-Reported Target Count for a total of *N* = 32 for analysis with this measure. A *p* < 0.05 was considered significant for all analyses and all analysis was conducted with IBM SPSS Statistics for Windows (Version 22, Armonk, NY: IBM Corp, Released 2013) software.

## Results

### Confirmation of Fixated Targets

On average, subjects reported the correct number of targets observed in the environment. A Related-Samples Wilcoxon Signed Rank Test compared the Self-Reported Target Count and the Gaze-Validated Target Count. There was no statistical difference between the two counts of the targets by subjects or identified by the system (*Z* = −0.573, *p* = 0.567). Median target counts were 15 for the Self-Reported and 14 for the Gaze-Validated.

### General Eye-Gaze Measurement Outcomes

On average, individual fixations had a median duration of about 0.30 s (300 ms) and a mean Fixation Rate of ~2.06 fixations-per-second throughout the main task when short fixations were removed. If short fixations were included (removal of the 100 ms cut-off threshold), the median duration decreases to 0.29 seconds (~4%). Therefore, we have determined that the removal of those fixations with a duration of less than 100 ms has a minimal effect on the individual duration of fixations outcome. Subjects looked at objects (e.g., motorcycle, dumpster, trail markers) in the virtual environment, with a Mean Number of Fixations of 7.1 and for a Mean Dwell Time of 2.60 s per each object. We found that fixations on the surrounding terrain and sky comprised, on average, about 47% of all fixations.

**Figure 6:**
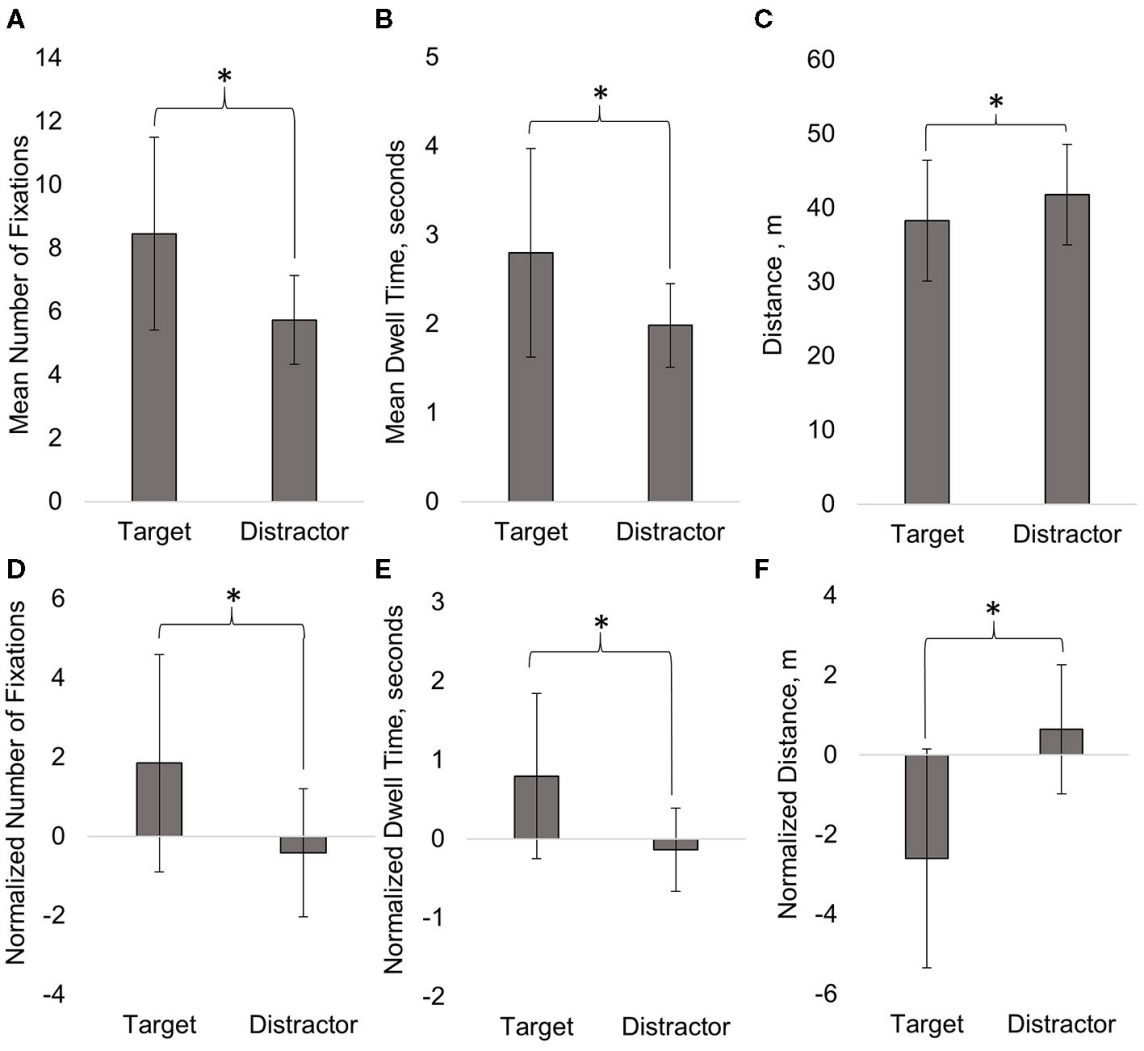
The Mean Number of Fixations **(A)**, Mean Dwell Time **(B)**, Mean Distance **(C)**, Normalized Number of Fixations **(D)**, Normalized Dwell Time **(E)**, and Normalized Distance **(F)** were significantly greater for targets compared to distractors. Mean ± SD (error bars) are shown on graph. *p<0.05.

Two separate one-way multivariate analysis of variances (MANOVAs) determined the effect of Fixation Object (target or distractor) on the normalized and non-normalized Mean Number of Fixations and Mean Dwell Time. There was a significant effect of Fixation Object for both non-normalized [*F*_(2,37)_ = 23.84, *p* = 0.000] and normalized gaze data [*F*_(2,36)_ = 22.54, *p* = 0.000; Figures 6A–C, D–F]. Two separate Univariate analysis of variances (ANOVAs) examined how Mean Number of Fixations and Mean Dwell Time differed depending on Fixation Object. Subjects significantly increased both the Mean Number of Fixations [*F*_(1,38)_ = 35.73, *p* = 0.000] and the Mean Dwell Time [*F*_(1,38)_ = 48.84, *p* = 0.000] for targets compared to distractors (Figures 6A,B). Two additional Univariate ANOVAs showed that Normalized Number of Fixations [*F*_(1,37)_ = 44.48, *p* = 0.000] and Normalized Dwell Time [*F*_(1,37)_ = 42.54, *p* = 0.000] also increased significantly for targets compared to distractors (Figures 6D,E). Mean Distance was compared between Fixation Objects using a Univariate ANOVA (Figure 6C). Subjects were significantly closer (less distance) to fixated targets compared to fixated distractors in the virtual environment [*F*_(1,37)_ = 12.99, *p* = 0.001]. A separate Univariate ANOVA showed that Normalized Distance [F_(1,38)_ = 18.53, *p* = 0.000] was also significantly less, on average, for targets (Figure 6F).

### Fixation Differences for the Humvee Condition and Motorcycle Condition

**Figure 7:**
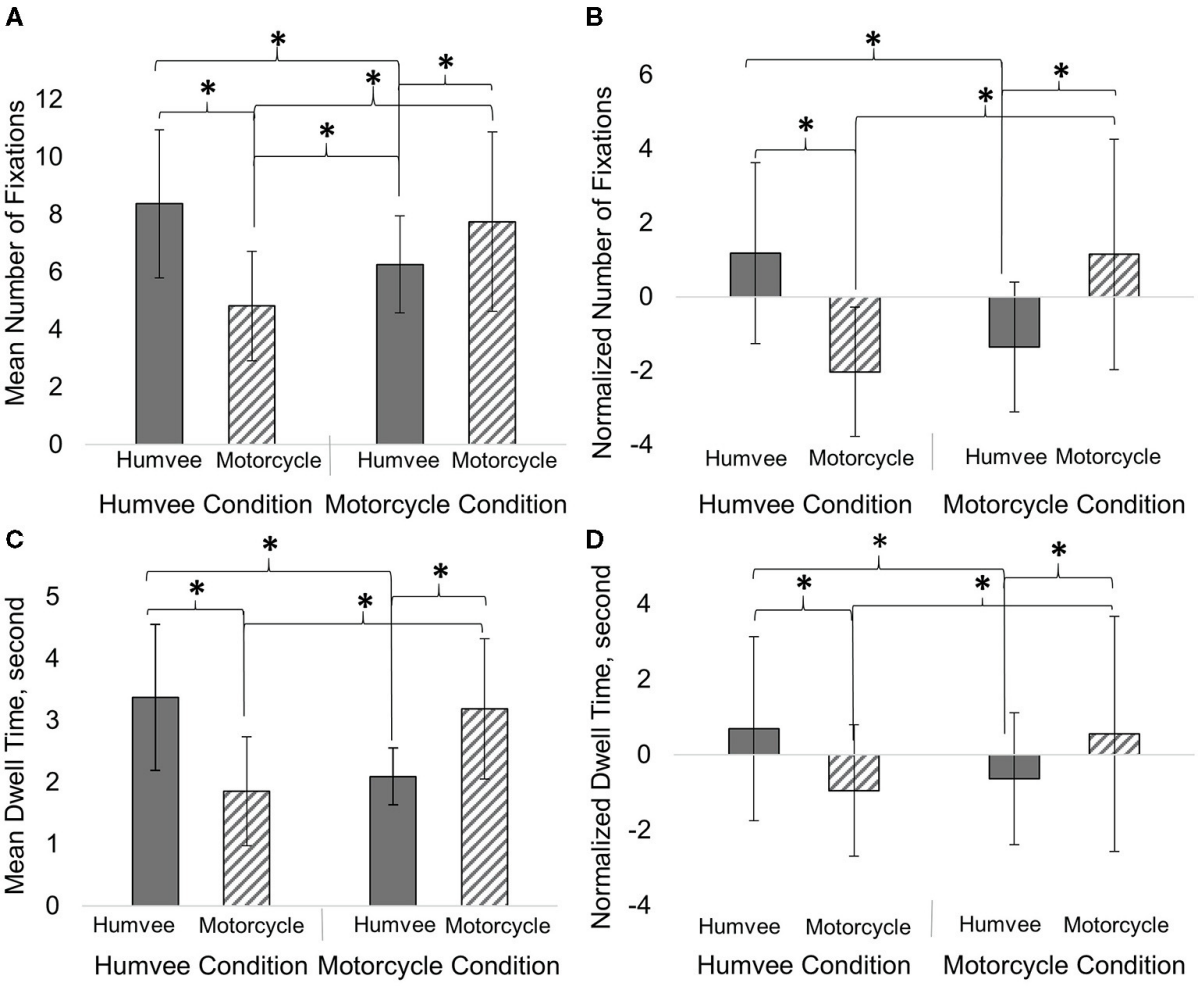
Both Target Conditions significantly increased Mean Number of Fixations **(A)**, Normalized Mean Number of Fixations **(B)**, Mean Dwell Time **(C)**, and Normalized Dwell Time **(D)** for their respective targets. Subject's increased visual attention toward their respective targets compared to the Fixation Object (a distractor). Mean ± SD (error bars) are shown on graph. *p<0.05.

Gaze data from the Humvee Condition and the Motorcycle Condition was used to quantify differences in the fixation metrics for the two target objects that were similar in terms of number, consistency, and dispersion along the path. Two separate two-way MANOVAs determined the effect of Target Condition and Fixation Object (limited to Humvees and motorcycles for this analysis) and the interaction of Target Condition and Fixation Object on the normalized and non-normalized Mean Number of Fixations and Mean Dwell Time. Overall, both non-normalized and normalized Mean Number of Fixations and Mean Dwell Time were significantly dependent upon the main effect of Fixation Object and the interaction between Target Condition and Fixation Object (Figure 7 and Table 2). Four separate Univariate ANOVAs determined that for the main effect of Fixation Object, only the Mean Number of Fixations (non-normalized) was significantly higher overall for the Humvee object compared to the motorcycle (Table 2). Four other separate Univariate ANOVAs determined that the interaction between Target Condition and Fixation Object was significantly different for non-normalized and normalized variables (Table 3). We found a significant main effect of Fixation Object for the Mean Number of Fixations, where there was an overall greater number of fixations for the Humvees compared to motorcycles (Table 3). Fixation Object was not a significant main effect for Mean Dwell Time, Normalized Number of Fixations, or Normalized Dwell Time. Tukey *Post-hoc* determined significant differences in those interactions. Both Target Conditions had significantly greater Mean Number of Fixations and greater Mean Dwell Time devoted to their targets, compared to the other object (*p* < 0.01, Tukey *Post-hoc*). Both Target Conditions had increased Mean Number of Fixations and Mean Dwell Time on their respective targets compared to that object for the other Target Condition (i.e., the Humvee Condition focused on the Humvees significantly more than the Motorcycle Condition focused on Humvees) (*p* < 0.01, Tukey *Post-hoc*). For these comparisons, the same pattern of *Post-hoc* analysis statistical significance was found for the Normalized Mean Number of Fixations and Normalized Dwell Time (*p* < 0.05). The Mean Number of Fixations for the Motorcycle Condition was significantly greater for the Humvee target compared to the Mean Number of Fixations for the Humvee Condition and the motorcycle target (*p* < 0.05, Tukey *Post-hoc*). No other differences were statistically significant.

**Table 2:** MANOVA for the non-normalized and normalized gaze data.

	**Wilks lambda** ***F*, *df***_**(2, 24)**_	***P*-value**	**Effect size, ***η***_*p*_^**2**^**
**Non-normalized gaze data (Mean number of fixations and mean** **dwell time)**			
Target condition	0.08	0.928	0.006
Fixation object	6.68	0.005^*^	0.357
Target condition × fixation object	19.64	0.000^**^	0.621
**Normalized gaze data (Normalized number of fixations and normalized dwell time)**			
Target condition	0.07	0.93	0.006
Fixation object	0.47	0.632	0.038
Target condition × fixation object	20.6	0.000^**^	0.632

** *p < 0.01,*

* *p < 0.05*.

**Table 3:** Univariate ANOVAs for the non-normalized and normalized gaze data.

	**Wilks lambda *F*, *df*_**(1, 25)**_**	***P*-value**	**Effect size, ***η***_*p*_^2^**
**Non-normalized gaze data (Mean number of fixations and mean**			
**dwell time)**			
**Mean number of fixations**			
Target condition	0.11	0.748	0.004
Fixation object	7.49	0.011^*^	0.22
Target condition × fixation object	38.56	0.000^**^	0.607
**Mean dwell time**			
Target condition	0.02	0.902	0.001
Fixation object	0.9	0.352	0.035
Target condition × fixation object	36.75	0.000^**^	0.595
**Normalized gaze data (Normalized number of fixations and normalized**			
**dwell time)**			
**Mean number of fixations**			
Target condition	0.14	0.707	0.006
Fixation object	0.62	0.438	0.024
Target condition × fixation object	41.38	0.000^**^	0.623
**Mean dwell time**			
Target condition	0.07	0.796	0.003
Fixation object	0.96	0.336	0.037
Target condition × fixation object	38.19	0.000^**^	0.604

** *p < 0.01,*

* *p < 0.05*.

### Effect of Cognitive Load

**Figure 8:**
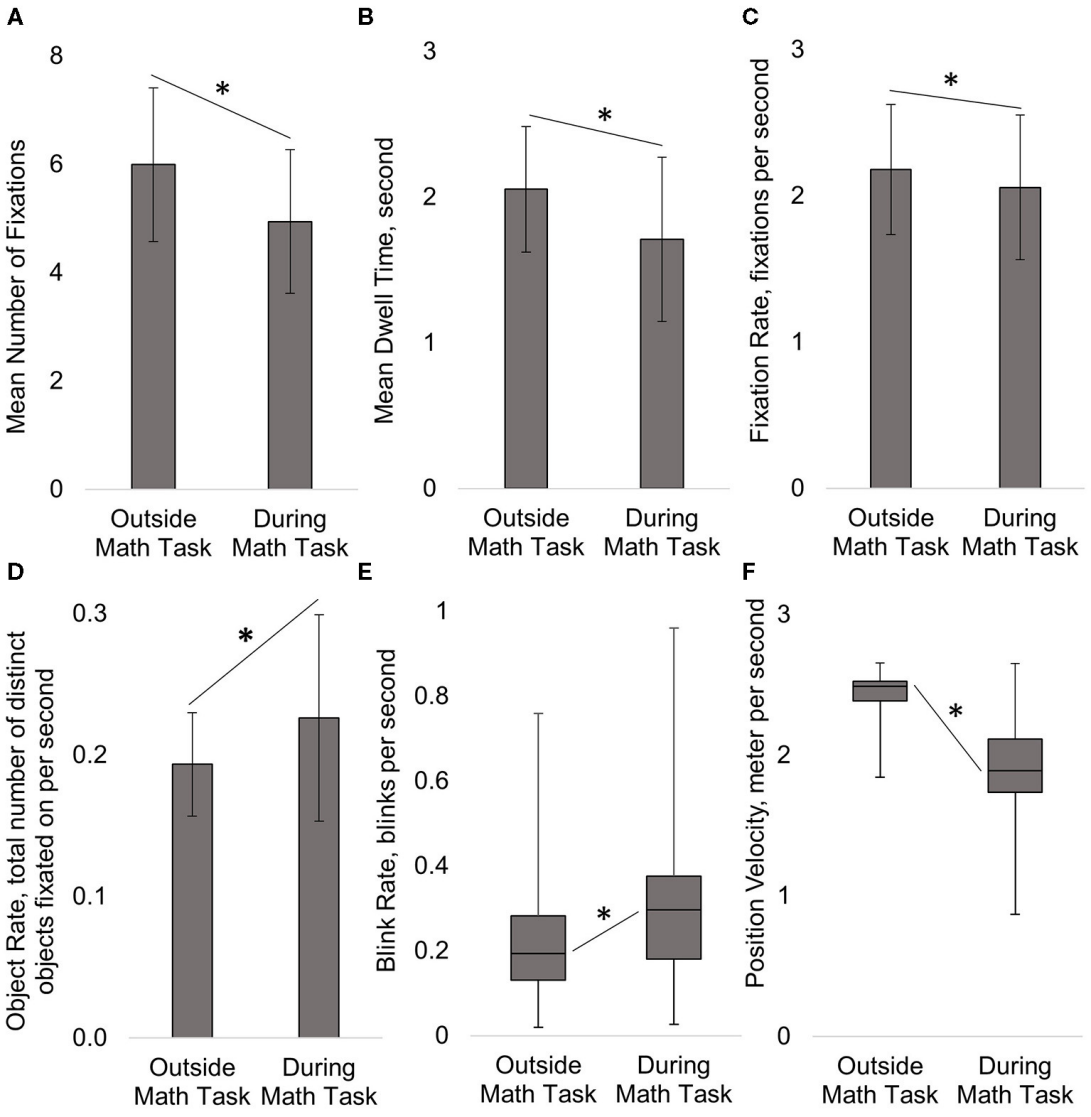
During the Math Task subjects significantly decreased the Mean Number of Fixations **(A)**, Mean Dwell Time **(B)**, and Fixation Rate on objects **(C)**, but increased the number of unique objects fixated on per unit of time, Object Rate **(D)** and Blink Rate **(E)**. Subjects significantly decreased their velocity navigating the environment **(F)** during the Math Task. The duration of individual fixations and the proportion of fixations on objects as opposed to terrain or sky, did not significantly change between time periods (not shown in figure). Mean ± Standard Deviation (error bars) shown on figures **(A–D)** and Median ± Interquartile Range (error bars) on figure **(E,F)**. *p<0.05.

The total time spent on the Math Task was ~150 s (~2.5 min), compared to the time spent outside of the Math Task (before and after) 713 s (~12 min). Paired samples *t*-test determined that subjects did not significantly change the Mean Duration of Individual Fixations *on objects* (*t*_36_ = 0.03, *p* = 0.979) during the Math Task compared to outside of the Math Task. However, Paired samples *t*-tests showed that subjects significantly decreased their Fixation Rate (*t*_37_ = −2.91, *p* = 0.006; Figure 8C). This discrepancy was explained by the relative increase in Blink Rate during the Math Task (Related-Sample Wilcoxon Signed Rank Test, *Z* = 3.78, *p* = 0.000; Figure 8E). There was also a significant reduction in the Mean Number of Fixations (*t*_37_ = −5.67, *p* = 0.000) *per object* and the Mean Dwell Time (*t*_36_ = −4.51, *p* = 0.000) *per object* during the Math Task, as compared to outside the Math Task (Figures 8A,B). In contrast, Object Rate increased significantly during the Math Task compared to outside the Math Task (*t*_37_ = 3.44, *p* = 0.001; Figure 8D). Interestingly, a Paired samples *t*-test showed that the Proportion of Fixations on Objects in the virtual environment (as opposed to fixations on terrain or sky) did not significantly change during the Math Task portion compared to outside of the Math Task (*t*_37_ = 0.16, *p* = 0.873). Additionally, a Related-Sample Wilcoxon Signed Rank Test showed that subjects significantly reduced their Position velocity, the speed at which they progressed through the environment, during the Math Task compared to outside the Math Task (*Z* = −4.87, *p* = 0.000; Figure 8F).

## Discussion

In this study, we demonstrate how an open-world, virtual environment can be used to identify task-relevant gaze behavior during navigation. Our approach enables us to collect meaningful, object-centered, gaze information during visual search in a cluttered landscape without restricting virtual head movement (i.e., camera position and orientation). Consistent with previous studies, we show a clear distinction in gaze behavior between target and distractor objects. Moreover, we quantify how this gaze behavior changes when subjects' attention is divided between visual search and secondary auditory task. Our results build on previous work using virtual environments (Karacan et al., 2010; Livingstone-Lee et al., 2011; Andersen et al., 2012; Draschkow et al., 2014; Jangraw et al., 2014; Kit et al., 2014; Li et al., 2016; Schrom-Feiertag et al., 2017; Olk et al., 2018; Clay et al., 2019; Helbing et al., 2020), extending the search space and incorporating a secondary task while maintaining the temporal and spatial precision needed for neurophysiological analysis.

### General Discussion

Here, we observed a direct link between gaze activity and specific objects within the virtual environment. Overall, subjects looked at targets significantly more often and longer than distractors. This confirms our initial hypothesis, based on previous studies in more restricted experimental contexts, and demonstrates the feasibility of gaze analysis in dynamic (constantly changing) environments. This study is unique in that we acquired data from a relatively large number of subjects (*N* = 39) navigating a detailed and complex virtual environment, but were still able to identify distinct condition-level gaze dynamics. This fixation and object-level precision enables meaningful inferences from the concurrent use of EEG (not reported here).

### Comparable General Outcomes to Literature

Overall, the basic eye movement outcomes were comparable to those found in literature with a few notable differences. We found that individual fixations had a duration of about 320 ms with a Fixation Rate of 2.06 fixations-per-second. This is comparable to a Foulsham et al. (2011), who found an average of 2 fixations-per-second and an individual fixation duration of 441 ms for subjects who watched a video of path they previously navigated. Subjects looked at objects (including targets and distractors) in the virtual environment, on average, about 7.1 times, comparable but higher than the number of Mean Number of Fixations reported by Zelinksy (2008) who found an average of 4.8 fixations to detect and locate military tanks in a realistic scene. In terms of dwell time, Clay et al. (2019) reported a Mean Dwell Time of 5.53 s on visual objects for subjects who freely navigated and observed houses (large objects) in a virtual town. This was higher than our reported Mean Dwell Time of 2.60 s per each object, perhaps due to the fact that the objects in our world were considerably smaller on average than the houses in the Clay et al. (2019) study and in that study subjects were freely exploring without searching for specific targets. Finally, ~47% of all our fixations were on the sky or terrain (path) surrounding the environment, which is comparably within range of what others have also noted on visual attention when walking. Foulsham et al. (2011) found that about 29% of fixations focused on the path ahead of where subjects were walking and Davoudian and Raynham (2012) who found about 50% of fixations were focused on the walking path. Overall, our general outcomes showed reasonable comparison to those found in previous work.

### Increased Number of Fixations and Dwell Time on Targets Compared to Distractors

In our study, we found subjects increased their visual attention (as measured by Mean Number of Fixations and Mean Dwell Time) on targets as compared to distractors. The Number of Fixations was significantly greater for targets compared to distractors. This 47% increase in fixations on targets over distractors was comparable to previous work. In a traditional visual search task with static images, Horstmann et al. (2019) found an approximate increase of 33% in Mean Number of Fixations for targets compared to target-similar distractors. Watson et al. (2019) found approximately a 10% increase overall in fixations for targets compared distractors in a study using a reward learning visual search task. We observed 41% increase in Mean Dwell Time for targets compared to distractors. This outcome was comparable to work by Draschkow et al. (2014) who observed around a 30% increase in Mean Dwell Time on targets compared to distractors during a timed visual search task of complex static naturalistic scenes. Our result, showing increased overt visual attention on targets, supports our claim that subtle changes in visual search behavior can be quantified in complex and dynamic virtual environments. Overall, our results were in line with previous studies, supporting the validity to our approach and processing methods.

It should be noted that the overall Mean Number of Fixations for both targets and distractors reported here, is greater than what has generally been found in many of the previous studies. This could be due to the task design and nature of the environment and task. Even though subjects were given a maximum time of 20 min to complete the task, subjects were not instructed to find their targets as quickly as possible, as is the case in many visual search studies. Thus, subjects had more time to visually inspect all objects in the environment, without feeling rushed. Our environment contained 15 targets for each condition and ~211 distractors (including other Target Conditions' targets, a ratio of targets to distractors of about 1:14). Increasing the number of search items or the number of distractors can impact the working memory load and reduce visual search efficiency (Palmer, 1995; Wolfe, 2007, 2012; Zelinsky, 2008; Gidlöf et al., 2013), especially in complex naturalistic environments (Wolfe, 1994a; Gidlöf et al., 2013). Therefore, the increased number of fixations observed in our study could be due to the subject's self-pace progression through the environment and the particular target to distractor ratio. Alternatively, movement through virtual environments generate a more diverse set of eye movements (e.g., smooth pursuit and optokinetic responses) which can impact the detection and labeling of ballistic saccades and inter-saccadic intervals (i.e., fixations).

Additionally, for some of our Target Conditions, target characteristics could have led to an overall high mean Number of Fixations on targets and distractors. For instance, distractors in some cases looked similar to the targets, especially at longer distances (i.e., Humvee vs. another large vehicle). The effect of target-distractor similarity could have led to the need for increased visual attention to confidently distinguish between targets and distractors and decreased search efficiency (Duncan and Humphreys, 1989; Wolfe, 1994b, 2007; Zelinsky, 2008; Horstmann et al., 2019). It should also be noted that novelty of an object could have increased frequency of fixations. For instance, we would expect to see a difference in the Mean Number of Fixations and Mean Dwell Time for the Aircraft Condition and the Furniture Condition who had targets that varied in characteristics and models compared to the Humvee Condition and Motorcycle Condition with a target that stayed the same throughout the environment and only change in position and orientation in the environment. Subjects with a variable target may have fixated on more objects in general to determine if they should be included in their target count. Previous work has shown a disproportionate increase in visual attention on distractors for searches involving multiple targets compared single, static targets (Menneer et al., 2012). Novelty of the target can increase the time it takes to identify the object as a target among (varied) distractors (Lubow and Kaplan, 1997). The effect of target variation was not assessed in the current report due to low subject recruitment numbers in Target Conditions with a varied target. However, similar to previous work with multiple targets, we would expect that those with variable targets may have heightened attention toward distractors, negatively impacting their visual search efficiency throughout the task. Target characteristics, such as target variation and target-distractor similarity, may have been contributing factors to the large Number of Fixations reported overall.

### Consideration of Contributing Factors Due to Task Design

Inherent differences in visual objects' shape, color, and size should have impacted visual attention toward specific objects in the virtual environment. However, rather than seeing these as limitations we argue that these are opportunities for additional, more nuanced research to better understand how: size, shape, color, visibility, context, etc. interplay with gaze behavior in ecologically valid environments. One would expect a greater Number of Fixations (and therefore greater Dwell Time) on the larger objects (e.g., Humvee, larger aircrafts, trucks, and buildings) compared to the smaller objects (e.g., furniture, motorcycles) due to being potentially visible at further distances. In contrast, a smaller object may be occluded by other larger objects or scenery until the subject is close to that object. In fact, we found that increased object surface size in the virtual environment was significantly and positively correlated with the Mean Number of Fixations, the Mean Dwell Time, and the Mean Distance from the object when the fixation occurred (see Materials and Methods). This was also evident in our additional analysis looking specifically how the Humvee and Motorcycle Conditions looked at Humvees and motorcycles. Overall, Humvees had significantly greater Number of Fixations compared to motorcycles. Furthermore, it is interesting to note that those in the Motorcycle Condition devoted a greater Number of Fixations to this large distractor object compared to what the Humvee Condition devoted to the smaller distractor object. Although there were a greater Number of Fixations devoted to the Humvee target overall, it is also interesting to note that there was not a significant difference in overall Mean Dwell Time. It appears that the duration of these additional fixations was rather short and, perhaps, unintentional or the object was not of real visual interest. Therefore, it could be that subjects naturally fixated more on the larger objects, even if such objects were not the target assigned to them and not relevant to their assigned task (Võ and Wolfe, 2012). This may have also given those assigned to Target Conditions with the larger targets, the Aircraft Condition and the Humvee Condition, a distinct advantage in seeing their targets due to visibility.

Along with size, visibility in terms of where the object was physically placed in the environment, may have also driven visual attention toward or away from some objects. Objects were sporadically placed throughout the environment and items placed at the end of long stretches of the path may have been central to subjects' attentional locus while navigating down the path toward trail markers. These items, especially ones that were centrally located along the horizontal plane, may have naturally drawn more visual attention (Karacan et al., 2010; Foulsham et al., 2011), especially if they were a larger object. For example, we found a surprisingly high number of fixations (~16.5 fixations) and dwell time (~5.9 s) on a particular GMC truck located at the end of a long canyon before a tight turn (compared to 7.1 fixations and 2.6 s averaged for all objects). When examined further, this particular object also had the highest Mean Distance (~107 m) compared to the overall Mean Distance all objects in the environment (~40 m). Therefore, some subjects could have fixated items due to their semi-random placement in the virtual environment rather than the due to the attributes of the item itself.

Scene context may also have impacted gaze toward certain objects in the virtual environment. For instance, the virtual environment was modeled as an arid and mountainous outdoor environment, but included some out of context items such as indoor furniture, musical instruments, a pool table, and a Ferris wheel. Scene context has shown to impact eye movement such as search time (Loftus and Mackworth, 1978; Henderson et al., 1999; Castelhano and Heaven, 2010) and memory recall (Draschkow et al., 2014). Items such as these may have garnered more visual attention due to their unexpected inclusion in the landscape (especially at the onset of the task) and/or could have been filtered as non-relevant visual objects if not assigned as a target that included those objects.

To help account for expected visual bias toward larger objects, random placement, or out of context objects in the virtual environment, we “normalized” each fixation metric for every object by subtracting the global mean for that object (the averaged value across all subjects for that particular object in the virtual environment). Normalization by simply dividing each gaze data point by the size of object (either 3D volume or 2D profile) in the virtual environment, resulted in a large bias toward the smaller targets. In contrast, our method of normalization enabled us to investigate object-centered gaze behavior for individuals compared to the mean across all conditions for any particular object. If in fact such bias was the cause of the increase in Mean Number of Fixations in the additional Humvee and Motorcycle Condition analysis for the Humvee object compared to the Motorcycle Condition, the normalization technique appeared to correct for such bias as differences were not present when using the Normalized Number of Fixations metric (Figure 7 and Table 3).

### Discrepancy With Virtual Environment and Real Life Walking Scenario in Distance of Focus

Mean distance in the virtual environment was around 40 m, with fixations on targets occurring at closer distances than distractors. As noted previously, our virtual environment allowed subjects to view objects down the path or to look around to their surroundings. Here, subjects appeared to fixate on objects relatively further away in their environment, which was previously noted for studies measuring gaze in a virtual setting (Clay et al., 2019). However, we would expect there to be some discrepancy between our findings and what occurs in real-world ambulation. Foulsham et al. (2011) found that people focus on objects further away in the view field when watching a first person video walking through an environment, compared to when they walked that environment in real life. In an ambulatory scenario, gaze is more often focused on near-field objects or terrain that could potentially affect gait. In a virtual environment navigation, gait perturbation is not a factor, thus near-field obstacles may be “under viewed” compared to what would occur in the real world.

### Effect of a Divided Attention Task on Gaze Data

During the Math Task, there was a significant shift in subjects' eye movement behavior resulting from the increase in cognitive load. We found that subjects focused on more objects per second during Math Task, not by increasing Fixation Rate or shortening duration of each individual fixation, but by decreasing the Mean Number of Fixations on each object and therefore, total time spent on processing each object. Subjects also slowed down their navigation speed (~24% decrease) and increased their Blink Rate (~46%) during the Math Task. Additionally, the Proportion of Fixations on Objects in the virtual environment as opposed to those fixations on terrain or sky did not change significantly when the auditory task was present. Therefore, subjects did not appear to alter their visual attention away from objects and drift toward more background items in the environment (terrain and sky). Together these results suggests that subjects appeared to compensate for increased cognitive load by reducing the object processing time, slowing their physical pace of progression through the environment, and increasing their Blink Rate.

The change in subjects' eye movement behavior are consistent with previous work showing a tendency to give attentional preference to auditory stimuli, potentially at the cost of one's visual processing capabilities (Robinson and Sloutsky, 2010; Dunifon et al., 2016) and an increase in Blink Rate (Magliacano et al., 2020). Neurophysiological work with EEG has shown that when auditory stimuli are paired with a visual task (cross-modal processing) there is a latency in the visual P300 response but no negative impact on the processing of auditory stimuli (Robinson et al., 2010). Buetti and Lleras (2016) found that when subjects were asked to complete an auditory math task while looking at a screen passively, that subjects showed a decreased response to visual events (appearance of an image) on the screen, suggesting a decreased sensitivity to visual events. These findings are consistent with the decrease in object processing (decreased Number of Fixations and Dwell Time) found in the current study. One reason we may have seen a decrease in the Number of Fixations during the Math Task, was that the number of blinks increased. The increase in Blink Rate is consistent with findings from Magliacano et al. (2020) where they found an increase in Blink Rate accompanying an auditory counting task with the absence of any visual task. Increased Blink Rate has also been found to coincide with visual scenes that require less attention and blinks are suppressed to reduce the chance of missing important information when visual attention is in demand (Nakano et al., 2009). Therefore, it is possible in our study that subjects disengaged from the visual task during the auditory math task, as evidence particularly by our significantly increased Blink Rate, due to the attention demand being comparatively low in the untimed visual search task. Due to the task design, we did not examine search efficiency in terms of a difference in fixations on targets and distractors during the Math Task, due to the auditory task occurring based on time in the environment (~8 min mark) and not physical place in the environment where target and distractor appearance could be controlled for all subjects.

Visual attentional demands during the task due appear to be important in attentional compensation strategy when an auditory math task is simultaneously introduced. When combining an auditory divided attention task with a visual mismatch detection task (find the mismatch as soon as possible), Pomplun et al. (2001) found reduced efficiency in completing the visual task when the auditory task was also present, seen as increased task reaction time (detection of mismatch), Number of Fixations and Dwell Time. Thus, the visual compensation strategy adopted when the auditory stimuli is present, may depend on the degree of continuous response required for the visual task at hand. Our findings may contradict those found by Pomplun et al. (2001) study, perhaps due low visual attentional demands required during our task compared to a more timed and speeded-response task. Our active navigation (exploratory and self-paced) visual search task required the identification of targets from distractor objects and only required subjects to continually identify and keep a mental count, not provide a continuous response within a tight time constraint. Thus, in our study, subjects could shift task priority from performance in the visual search task to the Math Task without any immediate negative consequence. However, verbal responses from some subjects post-study did indicate they were challenged in remembering multiple mental summations simultaneously (summation of the Math Task problems and keeping the target count) indicating that the co-occurrence of the Math Task with the visual search task did have an impact on cognitive load overall.

It should also be noted that our findings differ from previous work where cognitive workload was increased by adding to the difficulty of the visual task itself (with no auditory input). Others have found that as a visual task becomes more complex and difficult, there is an increase in the Mean Number of Fixations (King, 2009; Buettner, 2013; Zagermann et al., 2018), an increase in Dwell Time (duration of fixations) (King, 2009; Meghanathan et al., 2015), an increase in the number of saccades (Zelinsky and Sheinberg, 1997; Zagermann et al., 2018), an increase in saccade rate (Buettner, 2013), and a decrease in Blink Rate (Benedetto et al., 2011; Maffei and Angrilli, 2018) during the completion of that visual task. Therefore, how cognitive load is increased in the study design is, once again, important to consider when examining the effects of increased cognitive load on eye movement metrics. Overall, our findings provide additional insight into the effect of an additional auditory task during a self-paced visual search task in a natural virtual environment.

### Limitations

We would like to recognize several potential limitations to our study. One limitation was a restriction in data collection efforts due to public health concerns; we had to cease data collection earlier than planned and so were unable to have a balanced number of subjects in each Target Condition. This resulted in limited capabilities for comparison among the Target Conditions and their targets during the analysis. Second, while our experimental setup is similar to that of other studies, we utilized a desktop virtual environment instead of a virtual reality (VR) experience with a head mounted display. Although a VR system would provide a more immersive environment and allow for more free range in head and body movement compared to the current configuration, VR technology impose additional constraints when combining with other physiological measures, such as EEG. Likewise, simulator sickness is a common problem with immersive environments and our simulator sickness scores were relatively high overall. Simulator sickness could have impacted subject's natural viewing process through an environment and act as an unintended distractor from the task. Additionally, during the Math task it was observed that some subjects paused navigation when listening to the auditory number presentation (~1–5 s), contrary to instruction and encouragement from experimenters. Therefore, gazed behavior during this time would be a reflection of cognitive processing and not necessarily the visual search and navigation task. Furthermore, there was no auditory simulation provided outside of that provided during the Math Task. Therefore, differences in eye movement could also be attributed to simple auditory processing and not necessarily due to increased cognitive load from the Math Task itself. Therefore, future work in with this study design should include a passive auditory stimulation throughout the navigation to truly examine cognitive load effects on this virtual search task in this virtual environment. Finally, in the current study we did not investigate any temporal patterns in gaze metrics, such as the change in number of re-fixations and Dwell Time on targets and distractors over time as subjects progressed through the virtual environment. Such temporal patterns have previously been investigated when investigating efficiency during hybrid target search in static scenes (Drew et al., 2017). It may also be interesting to see how gaze metrics change temporally as a function of physical distance from the object in the environment (i.e., the distribution of fixations with respect to distance from the object). Such future work would provide a more complete picture of how subjects' search efficiency changed over time and space in the environment.

## Conclusion

In conclusion, we found that even during a self-paced navigation of a complex virtual environment, eye movement data can be used to robustly identify task-relevant gaze behaviors. There was a significant relationship between a subject's gaze behavior (Number of Fixations and Dwell Time), their Target Condition, and objects in the environment. When an additional auditory Math Task was introduced, subjects slowed their speed, decreased the Number of Fixations and Dwell Time on objects in the environment, increased Blink Rate, and increased the number of objects scanned in the environment. The present study adds to our understanding of how individuals actively search for information while navigating a naturalistic environment.

## Data Availability Statement

The raw data supporting the conclusions of this article will be made available by the authors, without undue reservation.

## Ethics Statement

The studies involving human participants were reviewed and approved by Institutional Review Board, ARL 19-122. The patients/participants provided their written informed consent to participate in this study.

## Author Contributions

LE was the primary individual on data processing, data analysis, and manuscript composition. SG also assisted in data processing. JT and SG contributed to the design and implementation of the original research. AR, JT, and SG contributed efforts and feedback on the analysis of the results and to the writing of the manuscript. RS wrote the software package for this study and provided feedback on the manuscript. All authors contributed to the article and approved the submitted version.

## Author Disclaimer

The views and conclusions contained in this document are those of the authors and should not be interpreted as representing the official policies, either expressed or implied, of the Army Research Laboratory or the U.S. Government. The U.S. Government is authorized to reproduce and distribute reprints for Government purposes notwithstanding any copyright notation herein.

## Conflict of Interest

LE, RS, and SG were employed by the company DCS Corp. The remaining authors declare that the research was conducted in the absence of any commercial or financial relationships that could be construed as a potential conflict of interest.

## Publisher's Note

All claims expressed in this article are solely those of the authors and do not necessarily represent those of their affiliated organizations, or those of the publisher, the editors and the reviewers. Any product that may be evaluated in this article, or claim that may be made by its manufacturer, is not guaranteed or endorsed by the publisher.
